# Nonprofessional Phagocytic Cell Receptors Involved in *Staphylococcus aureus* Internalization

**DOI:** 10.1155/2014/538546

**Published:** 2014-04-15

**Authors:** Nayeli Alva-Murillo, Joel Edmundo López-Meza, Alejandra Ochoa-Zarzosa

**Affiliations:** Centro Multidisciplinario de Estudios en Biotecnología-FMVZ, Universidad Michoacana de San Nicolás de Hidalgo, Km 9.5 Carretera Morelia-Zinapécuaro, CP 58893 La Palma. MICH, Mexico

## Abstract

*Staphylococcus aureus* is a successful human and animal pathogen. The majority of infections caused by this pathogen are life threatening, primarily because *S. aureus* has developed multiple evasion strategies, possesses intracellular persistence for long periods, and targets the skin and soft tissues. Therefore, it is very important to understand the mechanisms employed by *S. aureus* to colonize and proliferate in these cells. The aim of this review is to describe the recent discoveries concerning the host receptors of nonprofessional phagocytes involved in *S. aureus* internalization. Most of the knowledge related to the interaction of *S. aureus* with its host cells has been described in professional phagocytic cells such as macrophages. Here, we showed that in nonprofessional phagocytes the **α**5**β**1 integrin host receptor, chaperons, and the scavenger receptor CD36 are the main receptors employed during *S. aureus* internalization. The characterization and identification of new bacterial effectors and the host cell receptors involved will undoubtedly lead to new discoveries with beneficial purposes.

## 1. Introduction

*Staphylococcus *is a Gram-positive commensal and opportunistic human pathogen that causes serious community-acquired and nosocomial infections, including abscess formation, wound infection, endocarditis, osteomyelitis, pneumonia, and sepsis/septic shock [1, 2]. Additionally, strains of* S. aureus* cause diseases in cattle (mastitis), poultry, pigs, and horses [3, 4]. Treatment of these infections has become difficult because of the emergence of antibiotic-resistant strains [5].

Evidence exists that several strains of* S. aureus* have the ability to invade and persist within nonprofessional phagocytic cells (NPPCs), such as epithelial [6–8], endothelial [9, 10], osteoblast [11, 12], fibroblast [13, 14], and kidney cells [15, 16]. This ability enables the bacteria to evade the host innate immune system and to survive inside a wide variety of mammalian cells. Bacteria initially adhere to the cell membrane and extracellular matrix substrates through surface proteins (adhesins) [17, 18] and are then internalized by different NPPCs.

**Figure 1:**
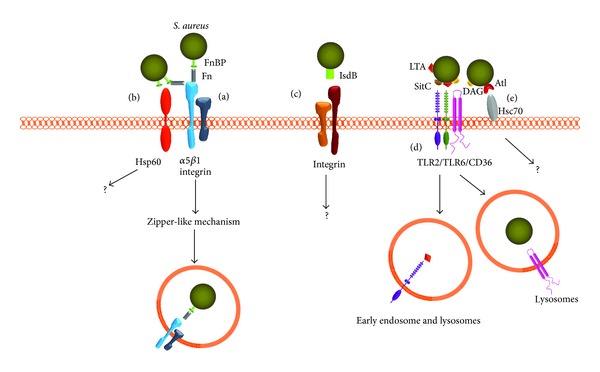
Different receptors and mechanisms involved in* S. aureus* internalization into nonprofessional phagocytic cells. (a) The first mechanism described for* S. aureus* internalization involved the *α*5*β*1 integrin host receptor and is mediated by bacterial FnBPs via Fn as a linking molecule; bacterial endocytosis is accomplished through a zipper-like mechanism [9, 16, 22, 23]. (b) FnBPs interact directly with host Hsp60 or with integrin and Hsp60 as a coreceptor through a Fn bridge [24], but the mechanism of endocytosis remains unknown. (c) The* S. aureus* iron-regulated surface determinant-B (IsdB) contributed to invasion, and IsdB most likely interacts with integrins that bind ligands with the RGD motif [25]; however, the endocytic pathway has not been determined. (d) TLR2 is involved in* S. aureus* internalization. CD36 acts as a coreceptor and is capable of recognizing diacylglycerides, whereas TLR2/TLR6 dimers recognize different PAMPs, such as LTA and SitC [26–28]. In monocytes TLR2 colocalizes with LTA in early endosomes and lysosomes [29]. In HeLa cells, internalized* S. aureus *colocalizes with CD36 [30]. (e) The host chaperone Hsc70 binds directly to autolysin (Atl) and mediates* S. aureus* internalization [31], but the endocytic routes remain uncharacterized.

Several reviews have discussed the intracellular persistence of this bacterium [19], the role of small colony variants (SCVs) [20], and the fate of the infected phagosome in professional phagocytes as well as in different NPPCs [21]. In this review, we will focus on the host NPPC receptors that are involved in the molecular interaction with* S. aureus *to accomplish bacterial internalization. Finally, we will discuss the medical implications derived from this knowledge and show a summary of the host receptors related to* S. aureus* internalization in NPPCs in Figure 1.

## 2. Bacterial Adhesion and Internalization

Bacterial internalization is a strategy that allows bacteria to evade the host immune response and to survive in the host cells. Several bacteria require initial adhesion to the host cell before the internalization process. Therefore, the adhesion and invasion into eukaryotic cells are major steps in bacterial pathogenesis [18].

Bacteria are capable of adhering to extracellular matrix components (i.e., collagen, vitronectin, fibrinogen, and especially fibronectin (Fn)) through protein-protein interactions mediated by “microbial surface components recognizing adhesive matrix molecules” (MSCRAMMs) or “secreted expanded repertoire adhesive molecules.” Additionally, bacterial adhesins recognize host cell surface elements such as integrins, cadherins, and selectins [18]. Pathogen adhesion occurs in two ways: (1) adhesins directly engage the host cell surface receptor, that is,* Listeria *spp. [37],* Yersinia* spp. [38, 39], and* Neisseria gonorrhoeae* [39, 40], and (2) bacterial connections form indirectly with the host receptor via the recruitment of extracellular matrix proteins (e.g.,* S. aureus*) [16, 41].

The bacterial engagement of eukaryotic receptors such as integrins often triggers a receptor-mediated internalization process that facilitates access to a protected intracellular niche, promoting bacterial replication [6, 42].

## 3. The Interaction between Nonprofessional Phagocyte Cell Receptors and* Staphylococcus aureus* Virulence Factors Promotes Internalization

*S. aureus* possesses a wide arsenal of virulence factors (adhesins, invasins, enzymes, toxins, and surface components) that contribute to the pathogenesis of infection (reviewed in Zecconi and Scali, 2013) [43]. These components promote the bacterial evasion of the host immune system as well as the colonization, dissemination, tissue damage, and transmission [1, 43].* S. aureus* expresses adhesins such as fibronectin-binding proteins (FnBPs), fibrinogen-binding proteins, elastin-binding proteins, collagen-binding proteins, clumping factor, extracellular adhesion protein (Eap), and protein A [43–45].* S. aureus* also possesses other cell-associated components such as capsular polysaccharide, peptidoglycan (PGN), and lipoteichoic acid (LTA) and secretes components such as enzymes (coagulase, lipase, hyaluronidase, and protease) and toxins (enterotoxins, toxic shock syndrome, hemolysins, and leukocidin), which are very important for the establishment of infection [1, 43, 46]. In the next sections, we will describe the* S. aureus* components and their cognate receptors in NPPCs that lead to bacterial internalization.

## 4. ***α*5*β*1** Integrin and Fibronectin Receptors

**Figure 2:**
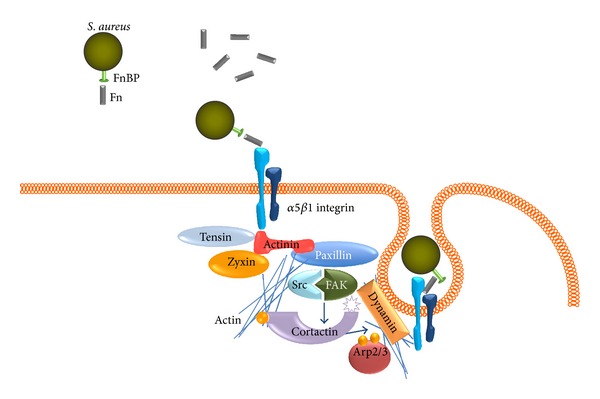
Summary of *α*5*β*1 integrin-mediated internalization of* S. aureus *into NPPCs. The RGD motif in fibronectin (Fn) is the crucial attachment site for fibronectin receptors, such as integrins. The activation and clustering of *α*5*β*1 integrin trigger particular signaling pathways and the accumulation of a focal adhesion-like protein complex in the vicinity of attached bacteria, as characterized by the recruitment of actinin, paxillin, zyxin, tensin, focal adhesion kinase (FAK), and Src kinase [32–34]. A crucial step in these signaling events is the reorganization of the actin cytoskeleton. Cortactin, an actin-binding protein, has been identified as one of the effectors of activated FAK and Src kinases, which associates with Arp2/3 complex to promote actin polymerization and binds to dynamin-2, a regulator of endocytosis [33, 35, 36].

Integrins are cation-dependent glycoprotein transmembrane receptors containing noncovalently associated *α*- and *β*- subunits [47]. In vertebrates, at least 18 *α*- and 8 *β*-subunits have been described [48]. Integrins have an extracellular binding domain that recognizes RGD or LVD sequences in ligands such as Fn, fibrinogen, vitronectin, and laminin [47, 48]. These receptors mediate a wide range of physiological and pathological processes, including cellular adhesion, migration, differentiation, apoptosis, phagocytosis, wound healing, and cancer. In addition, many integrins participate in pathogen recognition and host defense response in NPPCs; that is, *β*1 integrin mediates adhesion and endocytosis of* Yersinia *[39] and* S. aureus* [16, 41]. This event is mediated by a zipper-like process and depends on remodeling the actin cytoskeleton and membrane dynamics [49, 50]. The detailed mechanism for zipper-like-mediated internalization of* S. aureus* in NPPCs is shown in Figure 2.

Fn is a key dimeric glycoprotein in the extracellular matrix. The ability to bind to Fn is a characteristic of bacterial adhesion, which is a well-known mechanism described for many pathogens, including* S. aureus*. This bacterium expresses two closely related FnBPs encoded by the genes* fnbA* and* fnbB* [51], which are both contained in the majority of isolates with invasive properties [52].

Since the 1980s, it has been well recognized that* S. aureus *adhesion and internalization via a zipper-like process in NPPCs are mediated by integrins, Fn, and FnBPs. The role of FnBPs during* S. aureus* invasion has been established in endothelial cells [9, 10], osteoblasts [53], keratinocytes [54, 55], fibroblast [56], and epithelial cells [16, 22]. The events of internalization that occur via a zipper-like process were elucidated by experiments that included the following: (1) FnBP-deletion mutants of invasive strains; (2) noninvasive strains that express FnBPs; (3) the Fn-binding soluble domain isolated from FnBP; and (4) the blockage of receptors using anti-*α*5*β*1 or anti-Fn antibodies. The results of these approaches showed that FnBPA has a relevant role in invasion because its deletion in the* S. aureus *Cowan strain diminished the level of invasiveness (~80%) into a human embryonic kidney cell line (HEK 293) [16]. Similarly, an isogenic mutant (DU5883) of* S. aureus* (8325-4) that does not express FnBPs showed reduced internalization into transformed bovine mammary epithelial cells (MAC-T cells) [22], osteoblasts [53], and keratinocytes [57]. The role of FnBPs in host invasion was confirmed using complementation assays in which noninvasive strains transformed with plasmid overexpressing FnBPs were able to invade NPPCs [16]. The presence of FnBPs on the surface of* S. aureus* confers the advantage for tissue colonization* in vivo*, as observed in mammary glands, and confers the induction of severe infection [58, 59]. In addition, Dziewanowska et al. (1999) showed that FnBP-mediated bacterial uptake by NPPCs requires actin polymerization and is dependent on tyrosine kinases [22].

In contrast, the role of Fn was initially elucidated in HEK 293 cells. The preincubation of these cells with a soluble recombinant protein fragment composed of the Fn-binding domain of FnBP completely abolished the invasion by* S. aureus *Cowan and P1 strains, presumably by competing with the* S. aureus *FnBP to interact with the host cell receptor [16]. The use of polyclonal anti-Fn antibodies corroborated the role of Fn during* S. aureus* internalization in other cell types, for example, endothelial cells [9, 16, 24]. These data demonstrated that Fn mediates the interaction of* S. aureus* FnBPs with NPPCs.

The role of integrins during* S. aureus* internalization into NPPCs has been demonstrated by blockage experiments with antibodies. The blockage of integrin *α*5*β*1 by specific antibodies in HEK 293 [16], in HUVEC [60] cells, or in keratinocytes [57] demonstrated that these receptors have a relevant role during* S. aureus* internalization because their blockage leads to a significant reduction of internalized bacteria. Additionally, a monoclonal antibody specific for *β*1 integrins dramatically reduced* S. aureus* invasion into human Hep-2 cells [24]. In addition, a mutant mouse fibroblast line (GD25) lacking *β*1 integrin showed significantly reduced bacterial invasion [23]. Recent work by Ridley et al. (2012) showed that both the availability and functional state of integrin *α*5*β*1 are crucial for* S. aureus* invasion in different epithelial cells [61]. The use of GRGDS, a competitive inhibitor of *β*1 integrin ligands, has demonstrated the role of integrin during the internalization of* S. aureus* into alveolar epithelial cells (A549) by reducing the number of CFU recovered. In this work, the siRNA-mediated knockdown of *β*1 integrin expression in A459 cells significantly reduced* S. aureus* internalization (~50%) [8]. In addition, indirect evidence from our group established that the blockage of this integrin with latex beads covered with Fn inhibits* S. aureus* internalization into primary bovine mammary epithelial cells [62].

Overall, these results strongly suggest that* S. aureus* FnBPs and *α*5*β*1 integrin are necessary for efficient* S. aureus *internalization into NPPCs; however, other mechanisms are employed by this bacterium favoring its internalization that we will describe below.

## 5. Heat Shock Proteins 

Heat shock proteins (Hsps) are a group of evolutionarily highly conserved molecules that are expressed by prokaryotic and eukaryotic cells. These proteins perform important intracellular functions regarding protein folding and transport [63].

The role of Hsps during* S. aureus* internalization into NPPCs was first reported by Dziewanowska et al. (2000) [24]. Using a ligand blotting assay, Dziewanowska and colleagues identified that Hsp60 interacts with FnBP and showed that the pretreatment of epithelial cells with a monoclonal antibody specific for eukaryotic Hsp60 significantly reduces* S. aureus* internalization. Another Hsp related to* S. aureus* internalization in NPPCs is Hsc70. This protein is associated with viral infections by acting as a receptor for human T-cell lymphotropic virus type 1 (HTLV-1) [64] or rotaviruses [65, 66]. Hsc70 interacts with* S. aureus* hydrolases such as autolysin (Atl) during the bacterial internalization process. Atl participates in biofilm formation and mediates binding to the extracellular matrix and plasma proteins [31, 67, 68]. Hirschhausen et al. (2010) analyzed the* atl*-deficient* S. aureus* mutant SA113*atl* strain for its capability to be internalized into endothelial cells, and they showed the impaired ability of this strain to be endocytosed by these host cells [31]. Additionally, they reported that Atl binds directly to endothelial Hsc70 without a bridging molecule such as Fn. In addition, antibody blockade of Hsc70 decreases* S. aureus* internalization in these cells, and this protein has also been involved during* Brucella abortus* invasion into trophoblast giant cells [69], which suggests that this receptor is used as a generalized pathway during bacterial internalization.

## 6. Toll-Like Receptors 

TLRs offer an efficient and immediate response to bacterial, fungal, and viral infections by recognizing PAMPs. The TLR family consists of 13 mammalian members, and each member mediates an intrinsic signaling pathway and induces specific biological responses against microorganisms [70]. The cytoplasmic domain (Toll/IL-1 receptor domain) of TLRs is required for the signaling response leading to the activation of transcription factors such as NF-*κ*B [70]. The leucine-rich repeat (LRR) extracellular motif is responsible for the recognition of PAMPs [71]. TLRs are activated by ligand-induced multimerization and act by cooperating with several proteins such as other TLRs or coreceptors.

For* S. aureus* infections, TLR2 is the most relevant receptor involved in this process. TLR2 recognizes different PAMPs such as lipopeptides from Gram-positive and Gram-negative bacteria, lipoarabinomannan, LTA, PGN, atypical lipopolysaccharide, a phenol-soluble modulin from* S. epidermidis*, and others [72]. Additionally, TLR2 interacts with TLR1 and TLR6 in the process of ligand recognition, and the TLR2/TLR6 heterodimer recognizes the PGN in the macrophage phagosome [73] and a diacylated mycoplasma lipoprotein [74], while the TLR2/TLR1 heterodimer recognizes triacylated lipopeptides [75]. Reports have described the participation of TLR2 during* S. aureus* internalization in NPPCs; however, the results are not conclusive because TLR2 participation in phagocytosis may be indirect. For example, Rocha-de-Souza et al. (2008) indicated that TLR2 is involved in* S. aureus* internalization into human cord blood-derived mast cells using neutralizing antibodies [26]. The blockage of TLR2 in these cells decreases the number of bacteria internalized. In our work, we observed a similar result in primary bovine mammary epithelial cells (data unpublished); however, it remains to be clarified whether TLR2-mediated internalization is the consequence of the signaling activity of this receptor or whether the recognition of bacterial PAMPs by TLR2 is a key step for endocytosis. Although TLRs are not phagocytic receptors* per se*, they are also internalized in the process and participate in the link between phagocytosis and inflammatory responses by triggering the production of cytokines [76]. In addition, TLR2 is located in phagosomes and colocalizes with different* S. aureus *PAMPs. In NPPCs, the predominant triacylated lipoprotein of* S. aureus*, SitC, is located intracellularly with TLR2 in murine keratinocytes and stimulates proinflammatory cytokine expression [77]; however, SitC is internalized in a TLR2-independent manner. The results described above suggest that although no clear role of TLR2 has been observed during* S. aureus *internalization, this process appears to be a prerequisite for full TLR2 activation in both professional phagocytic cells as well as in NPPCs [76].

## 7. Coreceptors for TLR2 Mediate* Staphylococcus aureus* Recognition

CD36 is a membrane glycoprotein that belongs to the class B scavenger receptor family that interacts with other membrane receptors such as TLRs. This receptor plays a role during tumor growth, inflammation, wound healing, and angiogenesis and is able to recognize PAMPs or pathogen-infected cells by acting as a phagocytic receptor [78, 79]. During the host recognition of* S. aureus* mediated by TLR2, CD36 may act as a facilitator or coreceptor for diacylglyceride recognition through the TLR2/6 complex mediating bacterial invasion primarily in phagocytic cells [27]. In the NPPC line HEK 293, the overexpression of CD36 confers binding and uptake of* S. aureus*, suggesting a role for CD36 during the endocytosis of Gram-positive bacteria [28]. In addition, Leelahavanichkul et al. (2012) have demonstrated that intracellular* S. aureus* colocalizes with CD36 in HeLa cells [30]. CD14, a glycosylphosphatidylinositol-anchored membrane protein, is another coreceptor that participates in bacterial recognition by TLRs and enhances PGN and LTA signal transmission through TLR2 [80]. CD14/TLR2 is an essential receptor complex involved in Panton-Valentine leukocidin recognition [81]. CD14 and CD36 play a prominent role in LTA binding and enhancing LTA-induced signaling in human monocytes [29]. The aforementioned involvement suggests that CD14 may have a similar role as CD36 in* S. aureus* internalization; however, this effect remains to be fully explored.

## 8. Other* Staphylococcus aureus* Virulence Factors that Participate in the Internalization Process Interact with Uncharacterized Host Cell Receptors

As we have described above, several host receptors are used by* S. aureus* to invade NPPCs (Figure 1). Nonetheless, reports have indicated that different uncharacterized host receptors may be involved in* S. aureus* internalization in NPPCs. In the next section, we will describe several bacteria virulence factors involved in internalization whose host receptors remain to be characterized.

## 9. The Extracellular Adherence Protein

The extracellular adherence protein (Eap) in* S. aureus* binds to matrix extracellular components, inhibits leukocyte adhesion to endothelial cells, acts like an anti-inflammatory factor [82], and causes* S. aureus* agglutination [83]. This protein stimulates the adherence of* S. aureus *to epithelial cells [83] and fibroblasts [84]. Eap also participates during the bacterial internalization process because its absence reduces the adherence and internalization of* S. aureus* into fibroblast and epithelial cells [14], while the addition of exogenous Eap increases* S. aureus *internalization [85, 86]. This invasion process may be influenced by the 32 kDa neutral phosphatase that is located on the bacterial surface that binds to Eap [87]; however, no reports have yet described the identification of a host receptor for Eap.

## 10. Glyceraldehyde-3-Phosphate Dehydrogenase-C

Glyceraldehyde-3-phosphate dehydrogenase (GAPDH) is a glycolytic enzyme, and several GAPDH homologs present in bacteria are able to bind to Fn, lysozyme, plasminogen, and the cytoskeletal proteins myosin and actin. Therefore, this enzyme plays a role during* S. aureus* colonization and internalization [88, 89].* S. aureus* has two GAPDH homologs termed* gapA* (also known as* gapC* in a bovine mastitis isolate) and* gapB* [90], and both proteins are important in the pathogenesis of* S. aureus* in a* Galleria mellonella* model of infection [91].

*GapC* plays an important role during* S. aureus* internalization into MAC-T cells [92]. The number of CFUs recovered from an isogenic* gapC* mutant H330 strain that were adhered and internalized into MAC-T cells was lower than the number corresponding to the WT strain. Nevertheless, the absence of* gapC* does not completely abolish the attachment and internalization of the bacteria, which is most likely due to the presence of other bacterial adhesins [92]. No reports have yet described the identification of a host receptor that recognizes* gapC*.

## 11. Iron-Regulated Surface Determinant-B 

*S. aureus* acquires iron from host hemoglobin due to the bacterial expression of iron-regulated surface determinants (Isd) [93]. Zapotoczna et al. (2013) reported that iron-regulated surface determinant-B (IsdB) promotes the invasion of* S. aureus* into 293T and HeLa cells [25]. Additionally, they proposed that soluble* S. aureus *IsdB binds to and stabilizes the active conformation of integrins, enabling them to interact with RGD-containing ligands, which leads to bacterial internalization in an integrin-dependent pathway. In addition, IsdB adheres to platelets through the integrin receptor GPIIb/IIIa (aIIIbb3) [94]; however, this receptor has not been implicated in bacterial internalization.

## 12. Conclusions 

Phagocytosis is an essential component of innate and adaptive immune responses. In NPPCs, phagocytosis plays major roles in tissue maintenance, regeneration, and remodeling. However, pathogenic bacteria also employ many of the receptors involved in phagocytosis during the interplay between the host cell defense response and tissue colonization. Thus, phagocytosis, endocytosis, and intracellular trafficking can be exploited for therapeutic objectives such as intracellular drug delivery (for a wide and detailed description of these beneficial strategies, see Duncan and Richardson, 2012) [95]. In addition, the manipulation of the host cell membrane affects numerous events, including actin remodeling and phagocytosis. The characterization and identification of new bacterial effectors and the host cell receptors involved will undoubtedly lead to new discoveries with beneficial purposes. Many of the pathways operating during the intracellular trafficking of bacteria (e.g., autophagosome formation) may have roles in multiple pathologies such as cancer, metabolic diseases, or neurological disorders (reviewed in Rubinsztein et al. 2012) [96]. Furthermore, a very important role of integrins during apoptosis clearance has been established, which may be related to autoimmune disorders, atherosclerosis, cancer, or human age-related macular degeneration (reviewed in Sayedyahossein and Dagnino, 2013) [97]. All of these medical implications highlight the relevance of the study of phagocytic receptors in the infection of NPPCs by* S. aureus* (Figure 1) because diseases related to intracellular strains (e.g.,* S. aureus*) are chronic and recurrent, and many of them are life threatening.

## References

[1] Lowy FD (1998). Medical progress: *Staphylococcus aureus* infections. *The New England Journal of Medicine*.

[2] Sinha B, Fraunholz M (2010). *Staphylococcus aureus* host cell invasion and post-invasion events. *International Journal of Medical Microbiology*.

[3] Kerro Dego O, van Dijk JE, Nederbragt H (2002). Factors involved in the early pathogenesis of bovine *Staphylococcus aureus* mastitis with emphasis on bacterial adhesion and invasion. A review. *The Veterinary Quarterly*.

[4] Leonard FC, Markey BK (2008). Meticillin-resistant *Staphylococcus aureus* in animals: a review. *Veterinary Journal*.

[5] Lindsay JA (2010). Genomic variation and evolution of *Staphylococcus aureus*. *International Journal of Medical Microbiology*.

[6] Almeida PA, Matthews KR, Cifrian E, Guidry AJ, Oliver SP (1996). *Staphylococcus aureus* invasion of bovine mammary epithelial cells. *Journal of Dairy Science*.

[7] Garzoni C, Francois P, Huyghe A (2007). A global view of *Staphylococcus aureus* whole genome expression upon internalization in human epithelial cells. *BMC Genomics*.

[8] Wang JH, Zhang K, Wang N, Qiu XM, Wang YB (2013). Involvement of phosphatidylinositol 3-Kinase/Akt signaling pathway in beta1 integrin-mediated internalization of *Staphylococcus aureus* by alveolar epithelial cells. *Journal of Microbiology*.

[9] Peacock SJ, Foster TJ, Cameron BJ, Berendt AR (1999). Bacterial fibronectin-binding proteins and endothelial cell surface fibronectin mediate adherence of *Staphylococcus aureus* to resting human endothelial cells. *Microbiology*.

[10] Sinha B, Herrmann M (2005). Mechanism and consequences of invasion of endothelial cells by *Staphylococcus aureus*. *Thrombosis and Haemostasis*.

[11] Ellington JK, Reilly SS, Ramp WK, Smeltzer MS, Kellam JF, Hudson MC (1999). Mechanisms of *Staphylococcus aureus* invasion of cultured osteoblasts. *Microbial Pathogenesis*.

[12] Trouillet S, Rasigade J-P, Lhoste Y (2011). A novel flow cytometry-based assay for the quantification of *Staphylococcus aureus* adhesion to and invasion of eukaryotic cells. *Journal of Microbiological Methods*.

[13] Usui A, Murai M, Seki K, Sakurada J, Masuda S (1992). Conspicuous ingestion of *Staphylococcus aureus* organisms by murine fibroblasts *in vitro*. *Microbiology and Immunology*.

[14] Haggar A, Hussain M, Lönnies H, Herrmann M, Norrby-Teglund A, Flock J-I (2003). Extracellular adherence protein from *Staphylococcus aureus* enhances internalization into eukaryotic cells. *Infection and Immunity*.

[15] Murai M, Usui A, Seki K, Sakurada J, Masuda S (1992). Intracellular localization of *Staphylococcus aureus* within primary cultured mouse kidney cells. *Microbiology and Immunology*.

[16] Sinha B, François PP, Nüße O (1999). Fibronectin-binding protein acts as *Staphylococcus aureus* invasin via fibronectin bridging to integrin *α*5*β*1. *Cellular Microbiology*.

[17] Foster TJ, Höök M (1998). Surface protein adhesins of *Staphylococcus aureus*. *Trends in Microbiology*.

[18] Pizarro-Cerdá J, Cossart P (2006). Bacterial adhesion and entry into host cells. *Cell*.

[19] Garzoni C, Kelley WL (2009). *Staphylococcus aureus*: new evidence for intracellular persistence. *Trends in Microbiology*.

[20] Sendi P, Proctor RA (2009). *Staphylococcus aureus* as an intracellular pathogen: the role of small colony variants. *Trends in Microbiology*.

[21] Garzoni C, Kelley WL (2011). Return of the Trojan horse: intracellular phenotype switching and immune evasion by *Staphylococcus aureus*. *EMBO Molecular Medicine*.

[22] Dziewanowska K, Patti JM, Deobald CF, Bayles KW, Trumble WR, Bohach GA (1999). Fibronectin binding protein and host cell tyrosine kinase are required for internalization of *Staphylococcus aureus* by epithelial cells. *Infection and Immunity*.

[23] Fowler T, Wann ER, Joh D, Johansson S, Foster TJ, Höök M (2000). Cellular invasion by *Staphylococcus aureus* involves a fibronectin bridge between the bacterial fibronectic-binding MSCRAMMs and host cell *β*1 integrins. *European Journal of Cell Biology*.

[24] Dziewanowska K, Carson AR, Patti JM, Deobald CF, Bayles KW, Bohach GA (2000). Staphylococcal fibronectin binding protein interacts with heat shock protein 60 and integrins: role in internalization by epithelial cells. *Infection and Immunity*.

[25] Zapotoczna M, Jevnikar Z, Miajlovic H, Kos J, Foster TJ (2013). Iron-regulated surface determinant B (IsdB) promotes *Staphylococcus aureus* adherence to and internalization by non-phagocytic human cells. *Cellular Microbiology*.

[26] Rocha-de-Souza CM, Berent-Maoz B, Mankuta D, Moses AE, Levi-Schaffer F (2008). Human mast cell activation by *Staphylococcus aureus*: interleukin-8 and tumor necrosis factor alpha release and the role of toll-like receptor 2 and CD48 molecules. *Infection and Immunity*.

[27] Hoebe K, Georgel P, Rutschmann S (2005). CD36 is a sensor of diacylglycerides. *Nature*.

[28] Stuart LM, Deng J, Silver JM (2005). Response to *Staphylococcus aureus* requires CD36-mediated phagocytosis triggered by the COOH-terminal cytoplasmic domain. *The Journal of Cell Biology*.

[29] Nilsen NJ, Deininger S, Nonstad U (2008). Cellular trafficking of lipoteichoic acid and Toll-like receptor 2 in relation to signaling; role of CD14 and CD36. *Journal of Leukocyte Biology*.

[30] Leelahavanichkul A, Bocharov AV, Kurlander R (2012). Class B scavenger receptor types I and II and CD36 targeting improves sepsis survival and acute outcomes in mice. *Journal of Immunology*.

[31] Hirschhausen N, Schlesier T, Schmidt MA, Götz F, Peters G, Heilmann C (2010). A novel staphylococcal internalization mechanism involves the major autolysin Atl and heat shock cognate protein Hsc70 as host cell receptor. *Cellular Microbiology*.

[32] Agerer F, Michel A, Ohlsen K, Hauck CR (2003). Integrin-mediated invasion of *Staphylococcus aureus* into human cells requires Src family protein-tyrosine kinases. *The Journal of Biological Chemistry*.

[33] Agerer F, Lux S, Michel A, Rohde M, Ohlsen K, Hauck CR (2005). Cellular invasion by *Staphylococcus aureus* reveals a functional link between focal adhesion kinase and cortactin in integrin-mediated internalisation. *Journal of Cell Science*.

[34] Schröder A, Schröder B, Roppenser B (2006). *Staphylococcus aureus* fibronectin binding protein-A induces mobile attachment sites and complex actin remodeling in living endothelial cells. *Molecular Biology of the Cell*.

[35] McNiven MA, Kim L, Krueger EW, Orth JD, Cao H, Wong TW (2000). Regulated interactions between dynamin and the actin-binding protein cortactin modulate cell shape. *The Journal of Cell Biology*.

[36] Selbach M, Backert S (2005). Cortactin: an Achilles’ heel of the actin cytoskeleton targeted by pathogens. *Trends in Microbiology*.

[37] Pizarro-Cerdá J, Kühbacher A, Cossart P (2012). Entry of Listeria monocytogenes in mammalian epithelial cells: an updated view. *Cold Spring Harbor Perspectives in Medicine*.

[38] Isberg RR, Leong JM (1990). Multiple *β*1 chain integrins are receptors for invasin, a protein that promotes bacterial penetration into mammalian cells. *Cell*.

[39] Nägele V, Heesemann J, Schielke S, Jiménez-Soto LF, Kurzai O, Ackermann N (2011). Neisseria meningitidis adhesin NadA targets *β*1 integrins: functional similarity to *Yersinia invasin*. *The Journal of Biological Chemistry*.

[40] Van Putten JPM, Duensing TD, Cole RL (1998). Entry of OpaA+ gonococci into HEp-2 cells requires concerted action of glycosaminoglycans, fibronectin and integrin receptors. *Molecular Microbiology*.

[41] Hoffmann C, Ohlsen K, Hauck CR (2011). Integrin-mediated uptake of fibronectin-binding bacteria. *European Journal of Cell Biology*.

[42] Qazi SNA, Harrison SE, Self T, Williams P, Hill PJ (2004). Real-time monitoring of intracellular *Staphylococcus aureus* replication. *Journal of Bacteriology*.

[43] Zecconi A, Scali F (2013). *Staphylococcus aureus* virulence factors in evasion from innate immune defenses in human and animal diseases. *Immunology Letters*.

[44] Alexander EH, Hudson MC (2001). Factors influencing the internalization of *Staphylococcus aureus* and impacts on the course of infections in humans. *Applied Microbiology and Biotechnology*.

[45] Nguyen T, Ghebrehiwet B, Peerschke EIB (2000). *Staphylococcus aureus* protein A recognizes platelet gC1qR/p33: a novel mechanism for staphylococcal interactions with platelets. *Infection and Immunity*.

[46] Navarre WW, Schneewind O (1999). Surface proteins of gram-positive bacteria and mechanisms of their targeting to the cell wall envelope. *Microbiology and Molecular Biology Reviews*.

[47] Kumar CC (1998). Signaling by integrin receptors. *Oncogene*.

[48] Takada Y, Ye X, Simon S (2007). The integrins. *Genome Biology*.

[49] Hauck CR, Ohlsen K (2006). Sticky connections: extracellular matrix protein recognition and integrin-mediated cellular invasion by *Staphylococcus aureus*. *Current Opinion in Microbiology*.

[50] DeMali KA, Wennerberg K, Burridge K (2003). Integrin signaling to the actin cytoskeleton. *Current Opinion in Cell Biology*.

[51] Jonsson K, Signas C, Muller H-P, Lindberg M (1991). Two different genes encode fibronectin binding proteins in *Staphylococcus aureus*. The complete nucleotide sequence and characterization of the second gene. *European Journal of Biochemistry*.

[52] Peacock SJ, Day NPJ, Thomas MG, Berendt AR, Foster TJ (2000). Clinical isolates of *Staphylococcus aureus* exhibit diversity in fnb genes and adhesion to human fibronectin. *Journal of Infection*.

[53] Ahmed S, Meghji S, Williams RJ, Henderson B, Brock JH, Nair SP (2001). *Staphylococcus aureus* fibronectin binding proteins are essential for internalization by osteoblasts but do not account for differences in intracellular levels of bacteria. *Infection and Immunity*.

[54] Mempel M, Schnopp C, Hojka M (2002). Invasion of human keratinocytes by *Staphylococcus aureus* and intracellular bacterial persistence represent haemolysin-independent virulence mechanisms that are followed by features of necrotic and apoptotic keratinocyte cell death. *British Journal of Dermatology*.

[55] Edwards AM, Potter U, Meenan NAG, Potts JR, Massey RC (2011). *Staphylococcus aureus* keratinocyte invasion is dependent upon multiple high-affinity fibronectin-binding repeats within FnBPA. *PLoS ONE*.

[56] Hoffmann C, Berking A, Agerer F (2010). Caveolin limits membrane microdomain mobility and integrin-mediated uptake of fibronectin-binding pathogens. *Journal of Cell Science*.

[57] Kintarak S, Whawell SA, Speight PM, Packer S, Nair SP (2004). Internalization of *Staphylococcus aureus* by human keratinocytes. *Infection and Immunity*.

[58] Brouillette E, Grondin G, Shkreta L, Lacasse P, Talbot BG (2003). *In vivo* and *in vitro* demonstration that *Staphylococcus aureus* is an intracellular pathogen in the presence or absence of fibronectin-binding proteins. *Microbial Pathogenesis*.

[59] Shinji H, Yosizawa Y, Tajima A (2011). Role of fibronectin-binding proteins A and B in *in vitro* cellular infections and *in vivo* septic infections by *Staphylococcus aureus*. *Infection and Immunity*.

[60] Massey RC, Kantzanou MN, Fowler T (2001). Fibronectin-binding protein A of *Staphylococcus aureus* has multiple, substituting, binding regions that mediate adherence to fibronectin and invasion of endothelial cells. *Cellular Microbiology*.

[61] Ridley RA, Douglas I, Whawell SA (2012). Differential adhesion and invasion by *Staphylococcus aureus* of epithelial cells derived from different anatomical sites. *Journal of Medical Microbiology*.

[62] Gutiérrez-Barroso A, Anaya-López JL, Lara-Zárate L, Loeza-Lara PD, López-Meza JE, Ochoa-Zarzosa A (2008). Prolactin stimulates the internalization of *Staphylococcus aureus* and modulates the expression of inflammatory response genes in bovine mammary epithelial cells. *Veterinary Immunology and Immunopathology*.

[63] Tsan M-F, Gao B (2009). Heat shock proteins and immune system. *Journal of Leukocyte Biology*.

[64] Sagara Y, Ishida C, Inoue Y, Shiraki H, Maeda Y (1998). 71-kilodalton heat shock cognate protein acts as a cellular receptor for syncytium formation induced by human T-cell lymphotropic virus type 1. *Journal of Virology*.

[65] Guerrero CA, Bouyssounade D, Zárate S (2002). Heat shock cognate protein 70 is involved in rotavirus cell entry. *Journal of Virology*.

[66] Santana AY, Guerrero CA, Acosta O (2013). Implication of Hsc70, PDI and integrin alphavbeta3 involvement during entry of the murine rotavirus ECwt into small-intestinal villi of suckling mice. *Archives of Virology*.

[67] Biswas R, Voggu L, Simon UK, Hentschel P, Thumm G, Götz F (2006). Activity of the major staphylococcal autolysin Atl. *FEMS Microbiology Letters*.

[68] Artini M, Scoarughi GL, Papa R (2011). A new anti-infective strategy to reduce adhesion-mediated virulence in *Staphylococcus aureus* affecting surface proteins. *International Journal of Immunopathology and Pharmacology*.

[69] Watanabe K, Tachibana M, Tanaka S (2008). Heat shock cognate protein 70 contributes to Brucella invasion into trophoblast giant cells that cause infectious abortion. *BMC Microbiology*.

[70] Uematsu S, Akira S (2006). Toll-like receptors and innate immunity. *Journal of Molecular Medicine*.

[71] Akira S, Takeda K (2004). Toll-like receptor signalling. *Nature Reviews Immunology*.

[72] Akira S, Uematsu S, Takeuchi O (2006). Pathogen recognition and innate immunity. *Cell*.

[73] Ozinsky A, Underhill DM, Fontenot JD (2000). The repertoire for pattern recognition of pathogens by the innate immune system is defined by cooperation between Toll-like receptors. *Proceedings of the National Academy of Sciences of the United States of America*.

[74] Takeuchi O, Kawai T, Mühlradt PF (2001). Discrimination of bacterial lipoproteins by Toll-like recepttor 6. *International Immunology*.

[75] Takeuchi O, Sato S, Horiuchi T (2002). Cutting edge: role of Toll-like receptor 1 in mediating immune response to microbial lipoproteins. *Journal of Immunology*.

[76] Fournier B (2012). The function of TLR2 during staphylococcal diseases. *Frontiers in Cellular and Infection Microbiology*.

[77] Müller P, Müller-Anstett M, Wagener J (2010). The *Staphylococcus aureus* lipoprotein SitC colocalizes with toll-like receptor 2 (TLR2) in murine keratinocytes and elicits intracellular TLR2 accumulation. *Infection and Immunity*.

[78] Silverstein RL, Li W, Park YM, Rahaman SO (2010). Mechanisms of cell signaling by the scavenger receptor CD36: implications in atherosclerosis and thrombosis. *Transactions of the American Clinical and Climatological Association*.

[79] Baranova IN, Vishnyakova TG, Bocharov AV (2012). Class B scavenger receptor types I and II and CD36 mediate bacterial recognition and proinflammatory signaling induced by *Escherichia coli*, lipopolysaccharide, and cytosolic chaperonin 60. *Journal of Immunology*.

[80] Schwandner R, Dziarski R, Wesche H, Rothe M, Kirschning CJ (1999). Peptidoglycan- and lipoteichoic acid-induced cell activation is mediated by Toll-like receptor 2. *The Journal of Biological Chemistry*.

[81] Zivkovic A, Sharif O, Stich K (2011). TLR 2 and CD14 mediate innate immunity and lung inflammation to staphylococcal panton-valentine leukocidin *in vivo*. *Journal of Immunology*.

[82] Chavakis T, Hussain M, Kanse SM (2002). *Staphylococcus aureus* extracellular adherence protein serves as anti-inflammatory factor by inhibiting the recruitment of host leukocytes. *Nature Medicine*.

[83] Palma M, Haggar A, Flock J-I (1999). Adherence of *Staphylococcus aureus* is enhanced by an endogenous secreted protein with broad binding activity. *Journal of Bacteriology*.

[84] Hussain M, Haggar A, Heilmann C, Peters G, Flock J-I, Herrmann M (2002). Insertional inactivation of eap in *Staphylococcus aureus* strain Newman confers reduced staphylococcal binding to fibroblasts. *Infection and Immunity*.

[85] Hussain M, Haggar A, Peters G (2008). More than one tandem repeat domain of the extracellular adherence protein of *Staphylococcus aureus* is required for aggregation, adherence, and host cell invasion but not for leukocyte activation. *Infection and Immunity*.

[86] Bur S, Preissner KT, Herrmann M, Bischoff M (2013). The *Staphylococcus aureus* extracellular adherence protein promotes bacterial internalization by keratinocytes independent of fibronectin-binding proteins. *The Journal of Investigative Dermatology*.

[87] Flock M, Flock J-I (2001). Rebinding of extracellular adherence protein Eap to *Staphylococcus aureus* can occur through a surface-bound neutral phosphatase. *Journal of Bacteriology*.

[88] Modun B, Williams P (1999). The staphylococcal transferrin-binding protein is a cell wall glyceraldehyde-3-phosphate dehydrogenase. *Infection and Immunity*.

[89] Pancholi V, Fischetti VA (1992). A major surface protein on group A streptococci is a glyceraldehyde-3-phosphate-dehydrogenase with multiple binding activity. *Journal of Experimental Medicine*.

[90] Goji N, Potter AA, Perez-Casal J (2004). Characterization of two proteins of *Staphylococcus aureus* isolated from bovine clinical mastitis with homology to glyceraldehyde-3-phosphate dehydrogenase. *Veterinary Microbiology*.

[91] Purves J, Cockayne A, Moody PCE, Morrissey JA (2010). Comparison of the regulation, metabolic functions, and roles in virulence of the glyceraldehyde-3-phosphate dehydrogenase homologues gapA and gapB in *Staphylococcus aureus*. *Infection and Immunity*.

[92] Kerro-Dego O, Prysliak T, Perez-Casal J, Potter AA (2012). Role of GapC in the pathogenesis of *Staphylococcus aureus*. *Veterinary Microbiology*.

[93] Hazmanian SK, Skaar EP, Gaspar AH (2003). Passage of heme-iron across the envelope of *Staphylococcus aureus*. *Science*.

[94] Miajlovic H, Zapotoczna M, Geoghegan JA, Kerrigan SW, Speziale P, Foster TJ (2010). Direct interaction of iron-regulated surface determinant IsdB of *Staphylococcus aureus* with the GPIIb/IIIa receptor on platelets. *Microbiology*.

[95] Duncan R, Richardson SC (2012). Endocytosis and intracellular trafficking as gateways for nanomedicine delivery: opportunities and challenges. *Molecular Pharmaceutics*.

[96] Rubinsztein DC, Codogno P, Levine B (2012). Autophagy modulation as a potential therapeutic target for diverse diseases. *Nature Reviews Drug Discovery*.

[97] Sayedyahossein S, Dagnino L (2013). Integrins and small GTPases as modulators of phagocytosis. *International Review of Cell and Molecular Biology*.

